# Starvation ketosis following self-administered tirzepatide obtained via online services in a young woman later diagnosed with anorexia nervosa: a case report

**DOI:** 10.1186/s40337-026-01666-y

**Published:** 2026-06-04

**Authors:** Norio Yasui-Furukori, Kasumi Tasaki, Yusuke Kamiga, Yoshimasa Aso

**Affiliations:** 1 Department of Psychiatry, Dokkyo Medical University, Mibu, Tochigi Japan; 2 Department of Endocrinology and Metabolism, Dokkyo Medical University, Mibu, Tochigi Japan

**Keywords:** Tirzepatide, Starvation ketosis, Anorexia nervosa, GLP-1 receptor agonist, Self-medication, Eating disorder

## Abstract

**Background:**

Tirzepatide, a dual glucose-dependent insulinotropic polypeptide (GIP) and glucagon-like peptide-1 (GLP-1) receptor agonist, induces potent appetite suppression and substantial weight loss. Increasing access to incretin-based therapies through online services has raised concerns regarding metabolic and psychiatric complications, particularly in vulnerable individuals. We report a case of starvation ketosis associated with self-administered tirzepatide, followed by the clinical recognition of anorexia nervosa.

**Case presentation:**

A 21-year-old woman with no prior formal psychiatric or metabolic diagnoses had a 2-month history of dieting behaviors and intermittent binge-purge symptoms before tirzepatide initiation. Seeking further weight loss, she obtained tirzepatide through an online medical service and self-administered 2.5 mg weekly for approximately 4 weeks, followed by 5.0 mg weekly for another month, without direct medical supervision. She reportedly stated during the online consultation that she was overweight. Her body weight decreased from 47 kg to 41 kg (BMI 17.9 to 15.6 kg/m²). Approximately 2 months after initiation, she developed severe nausea, bilious vomiting, and presyncope, requiring emergency admission. Tirzepatide had been discontinued shortly before admission because of nausea. Laboratory evaluation revealed marked ketonemia (serum 3-hydroxybutyrate 2057 µmol/L), normoglycaemia (glucose 79 mg/dL), mildly elevated anion gap, normal HbA1c (5.1%), and no clinically significant acidaemia. She had no history of SGLT2 inhibitor use or habitual alcohol consumption. She was diagnosed with starvation ketosis and improved with intravenous glucose infusion and nutritional support. Shortly after discharge, psychiatric evaluation by a board-certified psychiatrist led to a diagnosis of anorexia nervosa, binge-eating/purging type. At early follow-up, body weight had increased to 42 kg, the Eating Attitudes Test-26 (EAT-26) score decreased to 14, but serum 3-hydroxybutyrate remained elevated at 166 µmol/L. At later outpatient reassessment several months later, body weight remained 42 kg, serum 3-hydroxybutyrate had normalized to 24 µmol/L, and the EAT-26 score was 16; however, her drive for thinness persisted.

**Conclusions:**

This case highlights that unsupervised tirzepatide use may be associated with starvation ketosis and the clinical recognition or exacerbation of eating-disorder psychopathology. Metabolic recovery did not parallel full improvement in eating-disorder symptoms, underscoring the need for careful screening, longitudinal psychiatric follow-up, and interdisciplinary management when incretin-based therapies are used.

## Introduction

Glucagon-like peptide-1 receptor agonists (GLP-1RAs) and dual GIP/GLP-1 receptor agonists have transformed the management of obesity and type 2 diabetes through potent appetite suppression, delayed gastric emptying, and marked weight reduction [1, 2]. Tirzepatide, a dual GIP/GLP-1 receptor agonist, has demonstrated superior weight reduction compared with semaglutide in a head-to-head phase 3b trial in adults with obesity [1].

Alongside their expanding clinical use, access to incretin-based agents through online services has raised concerns about use without adequate longitudinal supervision. Emerging reviews suggest that GLP-1RAs may improve, maintain, or worsen eating-disorder symptoms depending on the clinical context, underscoring the need for caution in vulnerable individuals [3–5]. Because these agents may substantially suppress appetite and reduce oral intake, severe caloric restriction and ketosis can occur in vulnerable individuals.

Starvation ketosis is a metabolic condition characterized by elevated ketone bodies resulting from prolonged caloric restriction, typically in the absence of hyperglycaemia or severe acidaemia [6]. In contrast, diabetic ketoacidosis is characterized by hyperglycaemia, increased ketones, and metabolic acidosis [7]. Here, we describe a case of starvation ketosis following unsupervised tirzepatide use, after which anorexia nervosa was clinically recognized.

## Case presentation

The patient was a 21-year-old woman with no documented formal psychiatric or metabolic disorders. There was no known family history of psychiatric disorders. According to the patient’s self-report, her body weight at age 18 years was 56 kg. She reported that, for approximately 2 months before tirzepatide initiation, she had engaged in dieting behaviors, including reducing meal frequency from three to two meals per day, as well as recurrent binge eating followed by self-induced vomiting. Because she had not undergone prior psychiatric evaluation, these symptoms were regarded as pre-existing disordered-eating behaviors rather than a previously established eating-disorder diagnosis. She had no habitual alcohol consumption.

In mid-2025, seeking additional weight reduction, she obtained tirzepatide (Mounjaro^®^) through an online medical service and initiated self-injection without direct medical supervision. During the online consultation, she reportedly stated that she was overweight; however, the full details of the information she provided were not available from the clinical record. The initial dose was 2.5 mg once weekly for approximately 4 weeks, after which the dose was increased to 5.0 mg once weekly. According to the patient’s self-report, her body weight at the start of tirzepatide administration (Month 0) was 47 kg. Over the subsequent 2 months, her weight decreased to 41 kg at a height of 162 cm, corresponding to a reduction in body mass index (BMI) from 17.9 kg/m² to 15.6 kg/m², representing an approximate 12.8% loss of body weight.

Approximately 2 months after initiating tirzepatide, she developed severe nausea, repeated bilious vomiting, and presyncope with difficulty maintaining an upright posture, which led to emergency admission. She discontinued tirzepatide shortly before admission because of the onset of severe nausea, and it was not resumed thereafter.

Laboratory investigations at admission revealed a markedly elevated serum 3-hydroxybutyrate level of 2057 µmol/L, indicating significant ketonemia. Plasma glucose was 79 mg/dL, HbA1c was 5.1%, and there was no history of SGLT2 inhibitor use. Serum electrolytes were as follows: sodium 141 mmol/L, potassium 3.8 mmol/L, and chloride 100 mmol/L. The anion gap was 16.9 mmol/L when calculated without potassium (20.5 mmol/L including potassium). Venous blood gas analysis demonstrated a pH of 7.437, partial pressure of carbon dioxide of 36.4 mmHg, bicarbonate of 24.0 mmol/L, and a base excess of 0.3 mmol/L, indicating the absence of clinically significant acidaemia. Serum lactate was 2.4 mmol/L, excluding clinically relevant lactic acidosis. In the context of marked ketonemia, normoglycaemia, mildly elevated anion gap, recent rapid weight loss, reduced oral intake, no habitual alcohol consumption, and no SGLT2 inhibitor exposure, she was diagnosed with starvation ketosis rather than diabetic ketoacidosis.

Treatment consisted of intravenous glucose-containing fluids and electrolyte replacement, followed by gradual reintroduction of oral intake. Her gastrointestinal symptoms and general condition improved, and she was discharged after several days of inpatient care. The clinical course is summarized in Table 1.

**Table 1:** Clinical time course of the patient

Time point (relative to tirzepatide initiation)	Body weight (kg)	BMI (kg/m²)	Tirzepatide dose	Key clinical features and findings
Age 18 years	56	21.3	None	Dieting behaviors; reduced meal frequency; recurrent binge eating with self-induced vomiting
Month 0	47	17.9	2.5 mg/week	Self-administration of tirzepatide obtained through online services; patient reportedly stated that she was overweight during the online consultation; no direct medical supervision
Month 1	—	—	5.0 mg/week	Continued appetite suppression and progressive weight loss
Month 2	41	15.6	5.0 mg/week	Severe nausea, repeated bilious vomiting, presyncope
Month 2 (emergency admission)	—	—	5.0 mg/week	Tirzepatide discontinued shortly before admission because of nausea; diagnosed with starvation ketosis
Month 2 (laboratory findings)	—	—	—	3-hydroxybutyrate 2057 µmol/L; glucose 79 mg/dL; HbA1c 5.1%; Na 141 mmol/L; K 3.8 mmol/L; Cl 100 mmol/L; anion gap 16.9 mmol/L without K (20.5 mmol/L with K); venous pH 7.437; PCO₂ 36.4 mmHg; HCO₃⁻ 24.0 mmol/L; base excess 0.3 mmol/L; lactate 2.4 mmol/L; no SGLT2 inhibitor use; no habitual alcohol consumption
Month 2 (discharge)	—	—	Discontinued	Symptoms improved after intravenous glucose infusion and nutritional refeeding
Month 2–3 (psychiatric evaluation)	41	15.6	None	Diagnosed by psychiatric clinical interview with anorexia nervosa, binge-eating/purging type; EAT-26 = 22
Early follow-up	42	16.0	None	3-hydroxybutyrate 166 µmol/L (reference range, 0–74); EAT-26 = 14
Later follow-up (several months later)	42	16.0	None	3-hydroxybutyrate 24 µmol/L (reference range, 0–74); EAT-26 = 16; drive for thinness persisted; ongoing psychiatric follow-up

*Abbreviations: BMI*,* body mass index; EAT-26*,* Eating Attitudes Test-26. Month 0 indicates tirzepatide initiation.*

Shortly after discharge, psychiatric evaluation was performed because of persistent anxiety related to eating and an intense fear of weight gain. At that time, her body weight remained 41 kg (BMI 15.6 kg/m²). Psychiatric assessment was conducted by a board-certified psychiatrist using a clinical interview, and the diagnosis was established according to DSM-5 criteria. Psychological assessment revealed a pronounced drive for thinness, body-image distortion, and ongoing eating-disorder psychopathology. Because binge eating and self-induced vomiting reportedly persisted after tirzepatide initiation, she was diagnosed with anorexia nervosa, binge-eating/purging type. The Eating Attitudes Test-26 (EAT-26) score was 22, exceeding the conventional cut-off score of 20 suggestive of an eating disorder.

Psychoeducation and nutritional counseling were initiated, and regular psychiatric follow-up was arranged. At early outpatient follow-up, her body weight had increased to 42 kg. Serum 3-hydroxybutyrate remained elevated at 166 µmol/L (reference range, 0–74 µmol/L), while the EAT-26 score had decreased to 14. At a later outpatient reassessment several months later, her body weight remained 42 kg (BMI 16.0 kg/m²). Serum 3-hydroxybutyrate had normalized to 24 µmol/L, whereas the EAT-26 score was 16. Although ketonemia had resolved, her drive for thinness persisted. She continues to receive outpatient psychiatric follow-up.

## Discussion

This case illustrates a clinically significant intersection of metabolic and psychiatric pathology: starvation ketosis temporally associated with unsupervised tirzepatide use in a young woman later diagnosed with anorexia nervosa. The case is notable not only because of the metabolic complication itself, but also because metabolic improvement did not parallel full psychiatric improvement.

### GLP-1/GIP receptor agonists and eating behavior

Tirzepatide exerts its effects through combined GIP and GLP-1 receptor agonism, producing marked appetite suppression and changes in ingestive behavior [8]. Although incretin-based therapies are effective for obesity treatment, reviews of GLP-1 receptor agonists in eating-disorder populations emphasize that these agents may improve, maintain, or worsen eating-disorder symptoms depending on patient characteristics and treatment context [3–5]. This bidirectional potential is especially relevant in patients with pre-existing disordered-eating behaviors or strong weight-related psychopathology.

Recent case reports describe semaglutide misuse in atypical anorexia nervosa, relapse of anorexia nervosa associated with tirzepatide, and other mental health effects associated with tirzepatide [9–11]. Pharmacovigilance data have suggested disproportionality signals for reported psychiatric adverse events associated with GLP-1 receptor agonists, including nervousness, stress, eating disorder, binge eating, fear of eating, and self-induced vomiting, although such data do not establish causality [12]. Taken together, these findings support the view that incretin-based therapies should not be considered purely metabolic agents, but rather drugs with potential psychobehavioral effects in susceptible individuals.

### Starvation ketosis without clinically significant acidemia

The present case is notable for marked ketonemia (serum 3-hydroxybutyrate 2057 µmol/L) without clinically significant acidaemia [6, 7]. The diagnosis of starvation ketosis was supported by normoglycaemia (79 mg/dL), normal HbA1c (5.1%), absence of SGLT2 inhibitor exposure or habitual alcohol consumption, and a mildly elevated anion gap, in addition to recent rapid weight loss and reduced oral intake. Starvation ketosis arises from glycogen depletion and enhanced fatty-acid oxidation during prolonged caloric restriction, typically with normal or low blood glucose levels. Unlike diabetic ketoacidosis, the acid-base disturbance may be mild or absent, particularly when assessment occurs early or after partial compensation.

In this patient, the combination of substantial recent weight loss, reduced oral intake, gastrointestinal symptoms, normoglycaemia, and marked ketonemia supported starvation ketosis rather than diabetic ketoacidosis [6, 7]. Clinicians should therefore consider measuring serum ketones in patients receiving incretin-based therapies who present with nausea, vomiting, malaise, or presyncope, even in the absence of overt acidaemia.

### Psychiatric implications

A central psychiatric implication of this case is that tirzepatide-associated appetite suppression and rapid weight loss likely interacted with pre-existing dieting behavior and may have contributed to the clinical recognition or worsening of eating-disorder psychopathology rather than unequivocal de novo onset of anorexia nervosa. The patient had engaged in dieting and binge-purge behavior before tirzepatide initiation, suggesting pre-existing vulnerability. However, the temporal association between self-administered tirzepatide, rapid weight loss, medical decompensation, and subsequent psychiatric diagnosis is clinically meaningful.

GLP-1 signaling influences reward-related and motivational pathways, and pharmacologically induced weight loss may function as a strong reinforcer for restrictive eating in vulnerable individuals [3, 5, 10]. In this context, the medication may have amplified pre-existing cognitive and behavioral tendencies centered on thinness, control, and fear of weight gain.

### Interpretation of EAT-26 scores during follow-up

The longitudinal EAT-26 findings in this case warrant cautious interpretation. Although the score decreased from 22 at initial psychiatric evaluation to 14 at early follow-up, and was 16 at later follow-up, these values should not be interpreted as evidence of remission. The EAT-26 was originally developed as a screening and case-finding instrument rather than a diagnostic tool [13]. The conventional threshold of 20 is intended to identify individuals who require further professional evaluation, but scores below 20 do not exclude clinically significant eating-disorder psychopathology. Moreover, a recent case-control study in Japan suggested that an optimal cut-off score for DSM-5 eating disorders may be lower, at 17 [14]. In the present case, therefore, the decline in EAT-26 scores likely reflects partial improvement in self-reported symptoms rather than full psychiatric recovery, particularly because the patient remained significantly underweight.

### Divergence between metabolic and psychiatric recovery

A particularly important feature of this case is that normalization of ketone levels did not coincide with full improvement in eating-disorder psychopathology. At early outpatient follow-up, serum 3-hydroxybutyrate remained elevated at 166 µmol/L despite an increase in body weight to 42 kg and a reduction in EAT-26 score from 22 to 14. At later follow-up, serum 3-hydroxybutyrate had normalized to 24 µmol/L, but her body weight remained 42 kg and her drive for thinness persisted; the EAT-26 score also remained elevated at 16. These findings suggest that metabolic stabilization after starvation ketosis does not necessarily indicate remission of the underlying eating disorder.

Clinicians should therefore interpret improvement in biochemical parameters or questionnaire scores cautiously and continue longitudinal psychiatric assessment, particularly in patients whose weight restoration remains limited and whose overvaluation of thinness persists.

### Societal and ethical considerations

The availability of GLP-1 products through online channels raises ethical and public-health concerns. Regulatory authorities have warned that unapproved GLP-1 products sold online may be counterfeit, mislabeled, of uncertain quality, or associated with dosing errors and adverse events [15]. Even when products are obtained through online medical services rather than illicit vendors, there may be insufficient psychiatric screening, inadequate nutritional assessment, and limited follow-up.

This case therefore has implications beyond the individual patient. It underscores the need for better education about the psychiatric risks of unsupervised weight-loss pharmacotherapy and for closer collaboration among psychiatrists, endocrinologists, and primary-care physicians. More rigorous pre-prescription screening, including verification of current body weight and BMI, assessment of eating-disorder symptoms and body-image concerns, review of psychiatric and family psychiatric history, and clearer longitudinal monitoring after prescription initiation, might have helped prevent this case. In patients with possible eating-disorder vulnerability, early referral to psychiatry and nutrition services should be considered. Recent clinical guidance has also emphasized careful prescribing, monitoring, and risk assessment when GLP-1 medications are used in individuals with eating disorders or eating-disorder vulnerability [16].

### Limitations

This case report has several limitations. First, disordered-eating behaviors were already present before tirzepatide initiation, and therefore causality cannot be definitively established. Second, detailed quantitative data on caloric intake, urine ketones, and serial acid-base measurements were limited. Third, the authenticity and storage conditions of the tirzepatide product obtained through online services could not be independently verified. Fourth, the full details of the information the patient provided during the online consultation were not available. Nevertheless, the temporal association between rapid weight loss, marked ketonemia, and subsequent psychiatric evaluation provides a clinically important illustration of the potential metabolic and psychiatric risks of unsupervised incretin use.

## Conclusions

Unsupervised tirzepatide use obtained through online services can be associated with serious metabolic complications such as starvation ketosis and may unmask or exacerbate pre-existing eating-disorder pathology. Importantly, metabolic recovery may precede psychiatric recovery. Careful screening, longitudinal psychiatric follow-up, and interdisciplinary management are therefore essential when caring for patients with disordered-eating vulnerability who use incretin-based therapies.

## Data Availability

All data relevant to this case report are included in the article.
